# Supplementary material for: Active site specificity profiling datasets of matrix metalloproteinases (MMPs) 1, 2, 3, 7, 8, 9, 12, 13 and 14
Source: Data Brief. 2016 Feb 22;7:299–310. doi: 10.1016/j.dib.2016.02.036 (PMC4777984; doi:10.1016/j.dib.2016.02.036)
Supplement: Supplementary file 10 — Supplementary material [file mmc10.zip › WebPICS_hMMP7_T_1%/P2prime.html]

 

PICS results


|  |  |
| --- | --- |
| **P2prime\_H**  9 in 291 sites   3.1 %    effects > 10 perc. pnts.  (vice-versa in brackets)  P2\_Q: 26.4 (11.9) |  |
| **P2prime\_I**  33 in 291 sites   11.3 %    effects > 10 perc. pnts.  (vice-versa in brackets)  P2\_E: 10.2 (10.6) |  |
| **P2prime\_M**  16 in 291 sites   5.5 %    effects > 10 perc. pnts.  (vice-versa in brackets)  P2\_M: 21.6 (34.5)   P3prime\_N: 14.3 (17.6) |  |
| **P2prime\_T**  18 in 291 sites   6.2 %    effects > 10 perc. pnts.  (vice-versa in brackets)  P1\_Q: 14.6 (12.0) |  |
